# Supplementary material for: Walking Speed Is Better Than Hand Grip Strength as an Indicator of Early Decline in Physical Function with Age in Japanese Women Over 65: A Longitudinal Analysis of the Tanno-Sobetsu Study Using Linear Mixed-Effects Models
Source: Int J Environ Res Public Health. 2022 Nov 27;19(23):15769. doi: 10.3390/ijerph192315769 (PMC9738335; doi:10.3390/ijerph192315769)
Supplement: Supplementary file 1 [file ijerph-19-15769-s001.zip › ijerph-2044215-supplementary.pdf]

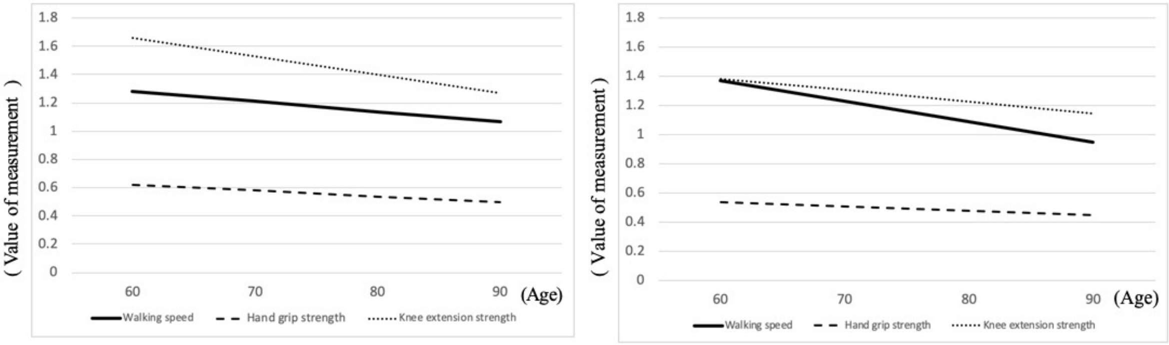

a; Men

b; Women

Figure S1. Age-related changes in actual measurements in the three physical function measures in men and women.
